# Self-Assembled Nanostructure of Ionic Sn(IV)porphyrin Complex Based on Multivalent Interactions for Photocatalytic Degradation of Water Contaminants

**DOI:** 10.3390/molecules29174200

**Published:** 2024-09-04

**Authors:** Nirmal Kumar Shee, Hee-Joon Kim

**Affiliations:** Department of Chemistry and Bioscience, Kumoh National Institute of Technology, Gumi 39177, Republic of Korea; nirmalshee@gmail.com

**Keywords:** Sn(IV)porphyrins, multivalent interactions, self-assembly, photocatalysts

## Abstract

[Sn(H_2_PO_4_)_2_(TPy*^H^*P)](H_2_PO_4_)_4_∙6H_2_O (**2**), an ionic tin porphyrin complex, was synthesized from the reaction of [Sn(OH)_2_TPyP] (**1**) with a dilute aqueous solution of a polyprotic acid (H_3_PO_4_). Complex **2** was fully characterized using various spectroscopic methods, such as X-ray single-crystal crystallography, ^1^H NMR spectroscopy, elemental analysis, FTIR spectroscopy, UV–vis spectroscopy, emission spectroscopy, EIS mass spectrometry, PXRD, and TGA analysis. The crystal structure of **2** reveals that the intermolecular hydrogen bonds between the peripheral pyridinium groups and the axially coordinated dihydrogen phosphate ligands are the main driving force for the supramolecular assembly. Simultaneously, the overall association of these chains in **2** leads to an open framework with porous channels. The photocatalytic degradation efficiency of methyl orange dye and tetracycline antibiotic by **2** was 83% within 75 min (rate constant = 0.023 min^−1^) and 75% within 60 min (rate constant = 0.018 min^−1^), respectively. The self-assembly of **2** resulted in a nanostructure with a huge surface area, elevated thermodynamic stability, interesting surface morphology, and excellent catalytic photodegradation performance for water pollutants, making these porphyrin-based photocatalytic systems promising for wastewater treatment.

## 1. Introduction

Porphyrin-based micro- and nanostructures have been especially appreciated by materials chemistry researchers due to their variable coordination features and interesting electronic and optical characteristics. They can be tremendously useful in several important applications, including photocatalysis for solar energy conversion [1], hydrogen production [2], carbon dioxide reduction [3], chemical sensors [4], photodynamic therapy for cancer treatments [5], the electrochemical oxygen evolution reaction [6], catalysis [7], and water purification [8]. This is because their nanoscale architectures exhibit a range of desirable characteristics, such as huge surface areas, interesting surface morphology, and outstanding chemical resistance, compared with their starting components. In particular, their substantial molecular design flexibility, exceptional photoelectronic property tunability, and solution-phase characteristics make them unique catalytic systems. Owing to their enormous absorption coefficients in the ranges of 400–440 nm (Soret band) and 500–720 nm (Q-bands), porphyrin derivatives are capable of absorbing light in a longer wavelength range (UV–vis) and have thus been used as photosensitizers for various photocatalytic systems [9,10,11,12,13,14]. Furthermore, their robust structural frameworks and inherent aromatic electronic characteristics encourage their large-scale self-aggregation in solution. A variety of intra- and intermolecular non-covalent interactions (e.g., hydrogen bonds, van der Waals forces, π–π stacking, hydrophobic/hydrophilic interactions, and electrostatic forces) drive the self-assembly of porphyrins [15,16,17,18,19,20]. Numerous self-assembly methods, such as re-precipitation [21], surfactant-assisted [22], metal–ligand coordination-assisted [23], and ionic [24] self-assembly, have been employed for the fabrication of porphyrin-based architectures. Ionic self-assembly methods are mainly used owing to their simple operation. In this process, oppositely charged species are electrostatically self-assembled to form nanoaggregates such as nanospheres [25], nanotubes [26], nanofibers [27], nanosheets [28], nanocomposites [29], and nanorods [30]. 

Among metalloporphyrins, Sn(IV)porphyrin compounds are ideal building block scaffolds for constructing coordination complexes [31,32], multiporphyrin arrays [33,34], and self-assembled nanostructures [35,36]. Oxophilic Sn(IV)porphyrin centers can readily accommodate two *trans-*axial oxyanion ligands (carboxylates and alkoxides), forming a rigid octahedral coordination geometry. Additionally, these complexes are diamagnetic, allowing structural details to be obtained from ^1^H and ^119^Sn nuclear magnetic resonance (NMR) spectroscopy [37]. Most importantly, the six-coordinate structure of Sn(IV)porphyrin complexes is advantageous for the self-assembly of tin porphyrin complexes because it allows the structural motifs to be extended into a multidimensional framework by utilizing various intermolecular interactions and different directions. Considering this structural feature, Sn(IV)porphyrin complexes can be recognized as molecular building blocks capable of multivalent interactions, which enable diverse and cooperative binding in supramolecular assemblies [38]. Previously, we crystallographically characterized the supramolecular ionic self-assemblies of highly charged tin(IV)porphyrin pyridinium cations stabilized by nitrate and sulfate anions [39,40]. These studies demonstrated that the axial ligands and counter anions of highly charged tin(IV)porphyrin cations play an important role in the supramolecular assembly together with the rigid structure of the porphyrin framework. In addition, Sn(IV)porphyrin cations assemble into different nanostructures depending on the counter anions and axial ligands [24]. The counter anions and axial ligands thus significantly control the morphology and photocatalytic properties of the nanostructures. From this perspective, we considered axial ligands and counter anions that may have multiple sites that can engage in intermolecular interactions, such as phosphate groups. The tetrahedral structure of the phosphate skeleton consisting of four oxygen atoms is useful for supramolecular assembly and the construction of robust nanostructures. Herein, we describe the synthesis, crystal structure, characterization, morphology, and catalytic performance of nanoaggregates of the Sn(IV)porphyrin-containing ionic complex [Sn(H_2_PO_4_)_2_(TPy^H^P)](H_2_PO_4_)_4_∙6H_2_O (**2**).

The synthesized porphyrin-based nanoaggregates were tested as visible-light catalysts for the decomposition of dyes in water. The catalytic performance of **2** for the photodecomposition of methyl orange (MO) dye and the antibiotic tetracycline (TC) was investigated. Additionally, the degradation kinetics and mechanism of the photocatalytic reaction are reported. The process is an environmentally friendly, cost-effective solution for wastewater treatment. As a highly water-soluble, aromatic azo dye, MO is generally used as a pH indicator in various industries, including printing, paper, food, and textiles. However, MO dye may be unethically discharged into water bodies, harming flora and fauna. It is one of 20 dyes identified most commonly in wastewater and can damage the skin, eyes, and respiratory system; moreover, it is non-biodegradable and carcinogenic, even at dilute concentrations. Owing to its hazardous nature, separating it from wastewater using common chemical, biological, and physical treatments is not straightforward. Previously, several researchers used their as-prepared catalysts to decompose MO dye in water and examined its catalytic efficiency [41,42,43,44,45,46,47,48,49,50,51,52,53,54,55]. TC, a phenanthrene core group-containing highly water-soluble antibiotic, exhibits broad-spectrum antimicrobial activity. TC is one of the most effective and cheapest medicines and finds a place on the list of essential medicines affiliated with the World Health Organization. It is extensively employed to cure malaria, cholera, pneumonia, typhus, syphilis, plague, and brucellosis. It also assists in curing chlamydia and rickettsia infections; combats Gram-positive/negative bacteria; and provides anabolic support for animals. It has a poor metabolic decay rate in water. Thus, the discharge of TC residues and their metabolites into water bodies significantly increases antibiotic resistance genes. Humans have experienced side effects such as diarrhea, rash, vomiting, kidney problems, poor dental development, and loss of appetite due to the existence of TC in drinkable water. TC has been used as a standard contaminant to evaluate the degradation efficiencies of as-synthesized catalysts [56,57,58,59,60,61,62,63,64,65,66,67,68,69,70,71]. In these studies, various investigations were conducted to verify the desirable requirements for the photodegradation of MO and TC pollutants in water.

## 2. Results and Discussion

### 2.1. Synthesis and X-ray Single-Crystal Structural Analysis

**Scheme 1:**
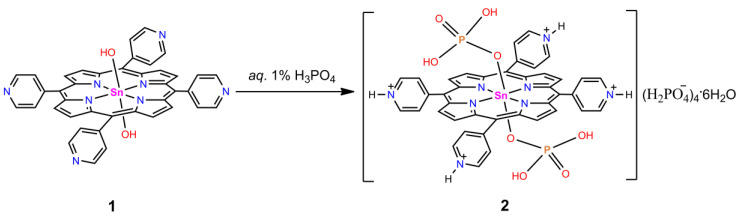
Synthesis of **2** from **1**.

The reaction of Sn(OH)_2_TPyP (**1**) with H_3_PO_4_ in a mixture of water and acetone affords crystalline [Sn(H_2_PO_4_)_2_(TPy^H^P)](H_2_PO_4_)_4_∙6H_2_O (**2**) (Scheme 1). The Materials and Methods Section 3 contains every detail of the synthesis procedure of **2** from **1**.

**Figure 1:**
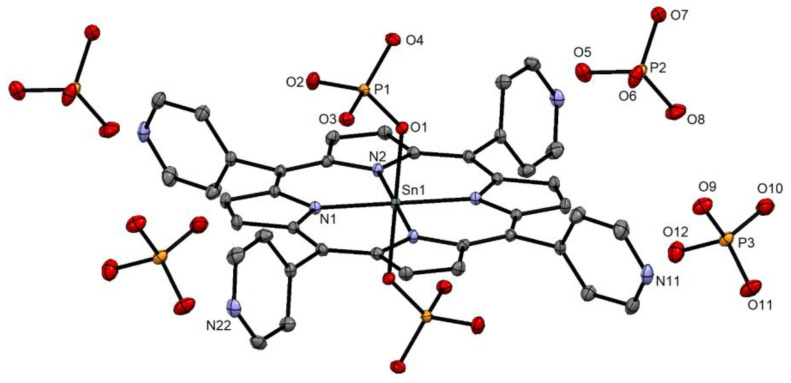
ORTEP diagram of **2** drawn at 50% probability level. All hydrogen atoms and solvents are excluded for clarity. Color code: cyan, Sn; yellow, P; gray, C; blue, N; red, O.

Rod-shaped violet single crystals of complex **2** were easily attained by the slow diffusion of acetone into a 1% aqueous H_3_PO_4_ solution of **1**. X-ray crystallographic data were accumulated after coating the single crystal of **2** with polymer glue. All structural refinement parameters and crystallographic data are given in Table S1, and selected bond lengths and bond angles are shown in Table S2. The crystal structure of **2** clearly shows the presence of Sn–O–PO(OH)_2_ bonds in the *trans*-axial position and four dihydrogen phosphate ions as counter anions (Figure 1). Although H_3_PO_4_ is a polyprotic acid, the H_2_PO_4_^–^ species (p*K*a_1_ = 7.5 × 10^−3^) is much more abundant than HPO_4_^2–^ (p*K*a_2_ = 6.2 × 10^−8^) and PO_4_^3−^ (p*K*a_3_ = 4.8 × 10^−13^) in aqueous solution.

**Figure 2:**
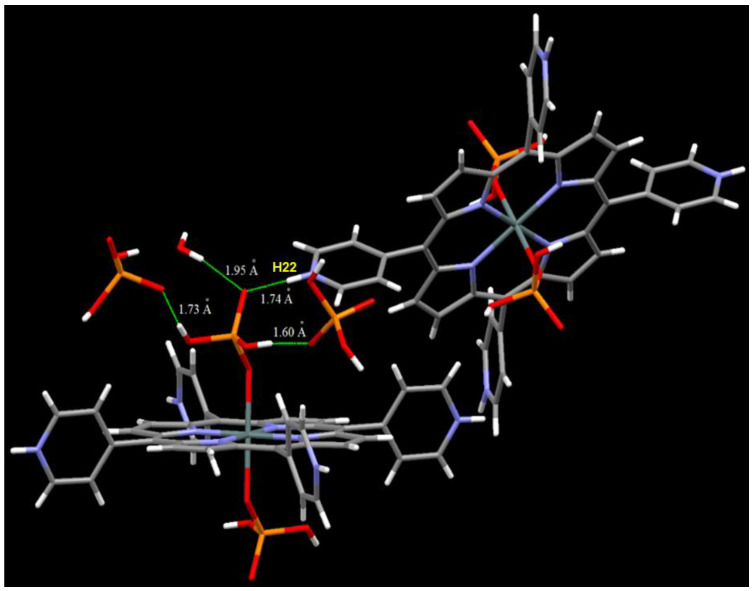
Intermolecular hydrogen bonds formed among the axial dihydrogen phosphate ligand in **2**, a protonated pyridyl substituent, H_2_PO_4_^−^ anion, and water molecule.

The reaction of **1** with an aqueous solution of 1% nitric acid has been reported to generate hexa-cationic [Sn(OH_2_)_2_TPy*^H^*P]^6+^ species surrounded by six nitrate anions [39]. In that case, the pyridine groups as well as the axial hydroxyl groups were protonated to produce [Sn(OH_2_)_2_TPy*^H^*P]^6+^. It is a hexa-cationic species and was stabilized by six NO_3_^−^ anions. In the unit cell of **2**, the axial hydroxyl group was replaced by the phosphoric acid group to form tetra-cationic [Sn(H_2_PO_4_)_2_(TPy^H^P)]^4+^ species and stabilized by four H_2_PO_4_^−^ anions. Also, six water molecules were present in the unit cell of **2**. The Sn(IV) atom in complex **2** has an octahedral coordination environment as evident from the crystal lattice. The equatorial plane and axial positions are occupied by four N atoms from the porphyrin moiety and two O atoms from phosphoric acid groups, respectively. Both positions of the axial phosphoric acid group bonded with the tin(IV)porphyrin center are symmetrically equivalent. The bond distances of two symmetrically comparable Sn−N bonds were measured to be 2.0837(15) Å and 2.0800(15) Å, respectively. The average bond distance is marginally shorter than that of the [Sn(OH_2_)_2_TPy*^H^*P]^6+^ species (2.083 Å). On the other hand, symmetrically equivalent, axial Sn−O bonds were measured to be 2.0838(13) Å. The average axial Sn–O bond length is considerably close to that of [Sn(OH_2_)_2_TPy*^H^*P]^6+^. The axial dihydrogen phosphate ligand plays a pivotal part in the supramolecular assembly of tetra-cationic [Sn(H_2_PO_4_)_2_(TPy^H^P)]^4+^. The dihydrogen phosphate ligand acts as both a hydrogen-bond donor and acceptor, as evident from Figure 2.

**Figure 3:**
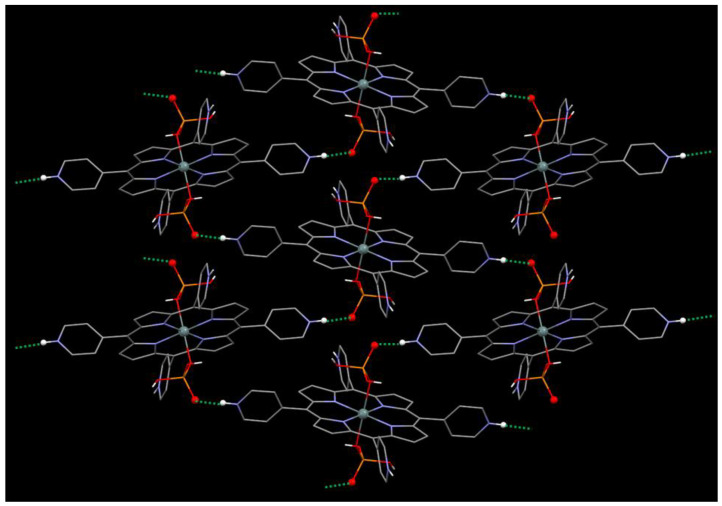
Hydrogen-bonded arrays of Sn(IV)porphyrin building blocks in **2**. The green dotted lines represent hydrogen bonds. For clarity, H atoms bonded to C atoms are excluded.

**Figure 4:**
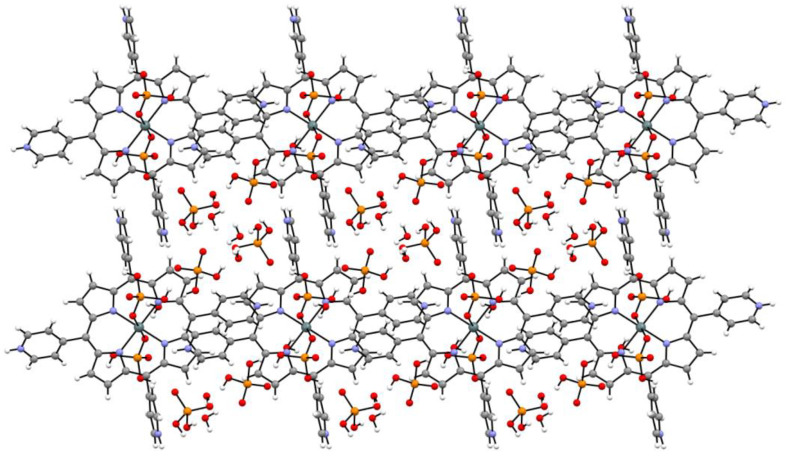
Supramolecular array of **2** including all species, viewed along the crystallographic *b*-axis.

**Figure 5:**
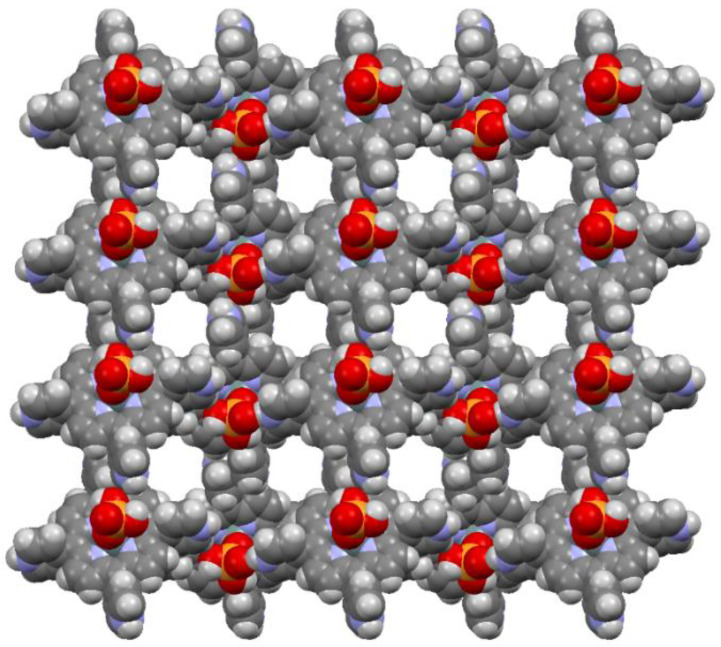
Space-filling representation showing the regular porous 1D channels in **2**, viewed along the crystallographic *b*-axis. The anions and lattice solvent molecules are excluded for clarity.

The axial dihydrogen phosphate ligand interacts with the pyridinium group of another porphyrin molecule. The hydrogen-bonded lengths from the axial O atom of the dihydrogen phosphate group to the N-pyridinium (N22−H22····O4) in the adjoining Sn(IV) porphyrin cation were found to be 1.740 Å (H22····O4) and 2.619 Å (N22····O4). In addition, the dihedral angle of N22−H22····O4 was measured to be 174.41^o^. Therefore, effective intermolecular hydrogen bonds give rise to two-dimensional association in **2** (Figure 3). Additionally, the axial dihydrogen phosphate ligand interacts with water molecules as well as two H_2_PO_4_^−^ counter anions, leading to the self-aggregation of **2**, as shown in Figure 4. The crystal structure of **2** reveals that the intermolecular hydrogen bonds between the peripheral pyridinium groups and the axially coordinated dihydrogen phosphate ligands are the main driving forces of supramolecular assembly. Simultaneously, the overall association of these chains in **2** results in an open framework with porous channels (Figure 5). The 1D chains in complex **2** do not interpenetrate. This leaves a void space in the framework that can be estimated by using the van der Waals surface method. The calculated free volume for complex **2** was found to be 42% per unit cell volume after removal of solvent molecules from the lattice (probe radius 1.3 Å and grid spacing 0.7 Å).

### 2.2. Spectroscopic Analysis

Complex **2** was comprehensively characterized using various instrumental techniques, including UV–vis spectroscopy, ^1^H NMR spectroscopy, elemental analysis, emission spectroscopy, Fourier-transform infrared (FTIR) spectroscopy, powder X-ray diffraction (PXRD), electrospray ionization mass spectrometry (ESI-MS), and thermogravimetric analysis. The ^1^H NMR spectrum of **2** in DMSO-d_6_ (dimethyl sulfoxide) is shown in Figure S1. A singlet at 9.57 ppm is assigned to the *β*-pyrrolic protons in **2**. The protons associated with the pyridyl rings appear as doublets at 9.12 ppm (for 3,5 positions) and 9.41 ppm (for 2,6 positions). In the case of **1**, these protons resonate at 9.12 ppm (s), 9.11 ppm (d), and 8.30 ppm (d) [72]. The spectra confirm that after complexation, the peak positions of these protons in **2** are downfield-shifted from those in **1** and have the same splitting patterns. The ESI mass spectrum of **2** is shown in Figure S2. The peak at *m*/*z* 233.67 (theoretical *m*/*z* of 233.52) corresponds to the molecular formula of [Sn(H_2_PO_4_)_2_TPy*^H^*P]^4+^. The peak at *m*/*z* 129.39 was designated as [Sn(OH_2_)_2_TPy*^H^*P]^6+^ (theoretical: 129.24). The experimental elemental (C, H, and N) data for **2** were in high agreement with the theoretical value.

**Figure 6:**
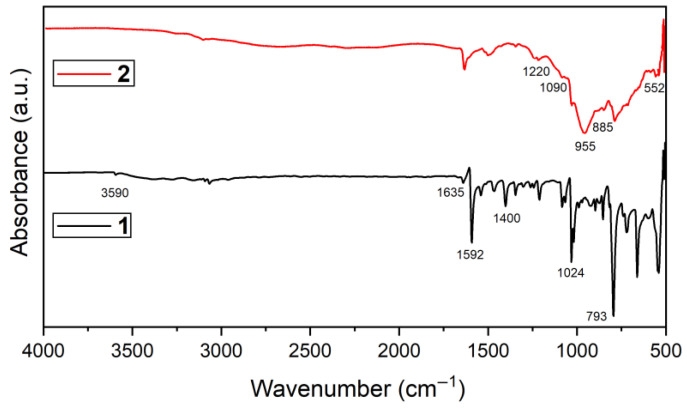
FTIR spectra of **1** and **2** (KBr).

Figure 6 compares the FTIR spectra of **1** and **2**. In the spectrum of **1**, the peaks at 1024 cm^–1^ and 793 cm^−1^ are attributed to the bending and out-of-plane bending vibration of C−H in the aromatic ring. The peaks at 1400 and 1592 cm^−1^ were designated to the stretching vibrations of C−N and C=C in the porphyrin ring, respectively. On the other hand, the peak at 1635 cm^−1^ is attributed to the stretching vibration of C=N in the porphyrin ring. The peak at 3590 cm^−1^ corresponds to the stretching vibrations of the axial OH group in **1**. In complex **2**, the peak at 3590 cm^−1^ associated with the O–H stretching band was absent owing to its complexation with H_2_PO_4_^−^. The X-ray single-crystal structure of complex **2** approves the absence of a Sn-OH bond. The characteristic P–O stretching and bending peaks of the H_2_PO_4_ moiety appear in the range of 1250–500 cm^−1^. These peaks are broad owing to the overlapping absorptions of the free H_2_PO_4_ ions and Sn-bonded H_2_PO_4_ ions in **2**. The peaks at 1220 and 1090 cm^−1^ are designated to the asymmetric stretching of the P–O bond, and those centered at 955 and 845 cm^−1^ are attributed to the symmetric stretching vibrations of the P–O bond; the peak at 552 cm^−1^ arises from the P–O bending vibration. All other peaks are identical to those of **1**. The above spectral data confirm the presence of dihydrogen phosphate anions in **2**.

**Figure 7:**
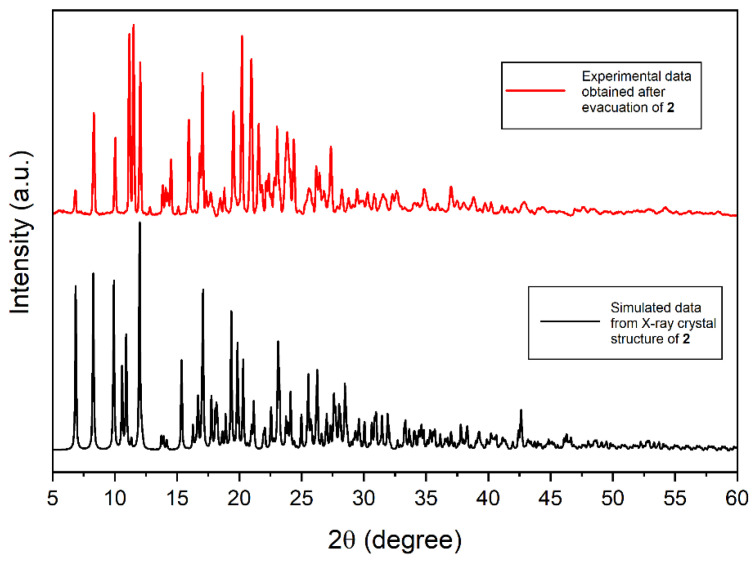
PXRD pattern of **2**.

The crystal structure of **2** contains six water molecules in the lattice. These solvent molecules seem to be vulnerable and may be lost from complex **2**. Therefore, the complex may lose its crystalline nature. To confirm the crystallinity of **2**, as-synthesized complex **2** was heated at 90 °C for 8 h in vacuum. The measured PXRD pattern of **2** in the 2θ range of 5–60° is shown in Figure 7. For comparison, this pattern is contrasted with the simulated PXRD pattern of 2 derived from the X-ray single-crystal structure. Complex **2** maintained a highly crystalline nature with a primary match between the experimental and simulated, as indicated by the powder-XRD patterns of evacuated samples of **2** (Figure 7). Both PXRD patterns were almost similar in the number of peaks, position, and relative intensities. This demonstrates the high rigidity and robustness of the porphyrin-based framework in **2**. The structure of the framework remains intact even after the eviction of the solvents.

The Brunauer–Emmett–Teller (BET) surface areas of as-prepared **1** and **2** were evaluated by N_2_ adsorption–desorption at 77 K to measure their changes in surface area (Figure S3). In Figure S3, compound **1** clearly shows a type-I isotherm, whereas complex **2** displays a type-IV isotherm. The BET surface area of **1** (26 m^2^/g) was found to be lower than that of **2** (92 m^2^/g). Thus, a remarkable increase in surface area was observed owing to the transformation of **1** into **2**. It is noteworthy that high mesoporosity has a positive impact on the adsorption of pollutant dyes.

**Figure 8:**
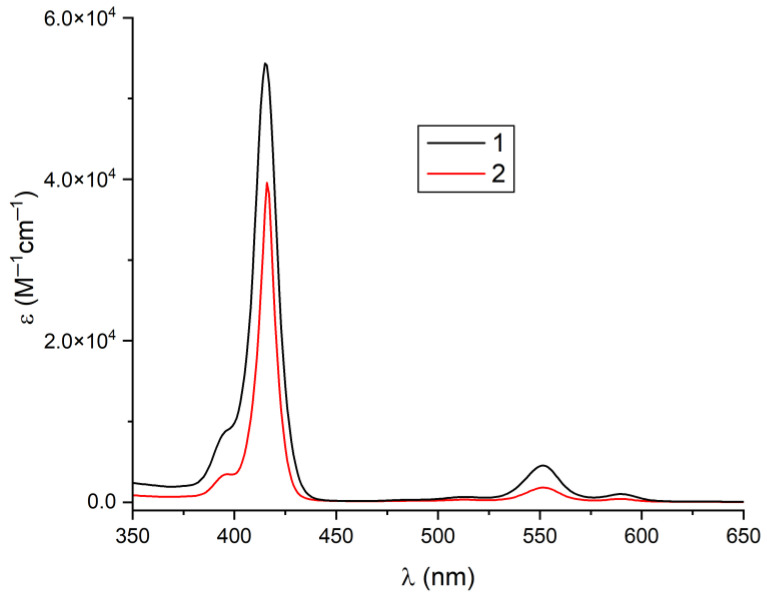
UV–visible spectra of **1** and **2** in DMSO.

Both the solid- and solution-phase optoelectronic properties of **1** and **2** were analyzed using UV–vis spectra. In Figure 8, **2** exhibits a sharp band at 416 nm (the Soret band), and three weak peaks at 512, 550, and 590 nm (Q-bands). This absorption spectrum was similar to that of **1**, in which these absorptions appeared at 415, 512, 550, and 589 nm, respectively. This confirms that **2** remains stable in solution after complexation of **1** with H_3_PO_4_. In the solid-phase UV–vis spectrum, **2** exhibits a broad Soret band at 432 nm (half-width center at 440 nm) and Q-bands in the range of 540 nm to 650 nm (Figure S4). In the spectrum of **1**, these peaks appeared at 428, 568, and 608 nm.

**Figure 9:**
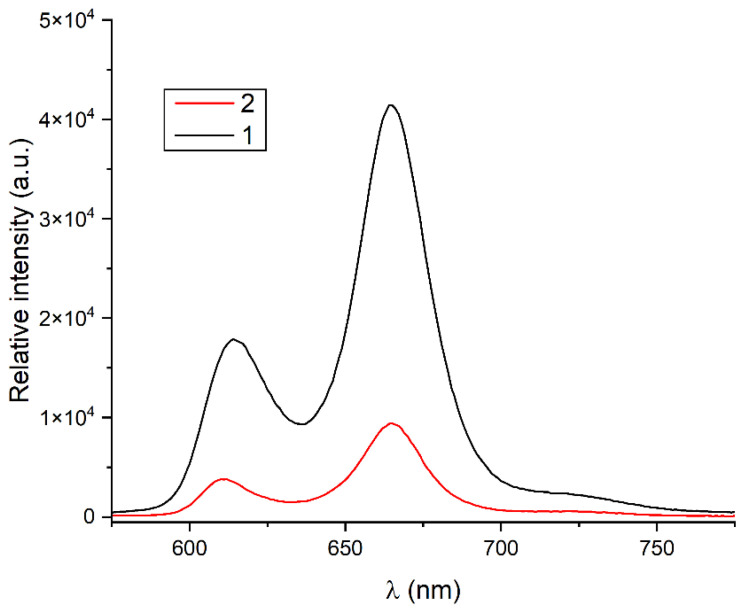
Fluorescence spectra of **1** and **2** in DMSO (λ_ext_ = 550 nm; optical density = 0.3).

The emission spectra of **1** and **2** in DMSO solvent are shown in Figure 9. The emission spectrum of **1** displays two-band fluorescence peaks at 614 and 665 nm. The photoluminescence spectrum of **2** also displays two-band fluorescence peaks at 610 and 664 nm. The peak-to-peak intensity ratios of **1** and **2** are also changed. From the spectral data, we confirmed that after complexation, the fluorescence intensity of **1** was quenched drastically in **2** (Figure 9).

Thermogravimetric analysis (TGA) was used to examine the thermal stability of **2** (Figure S5). When heated in the range of 50 to 100 °C, **2** lost ≈10% of its weight owing to the ousting of clathrate H_2_O molecules. The architectures of **2** appeared to be thermally robust up to ≈400 °C. Afterward, considerable weight loss was observed due to the breakdown of porphyrin frameworks.

**Figure 10:**
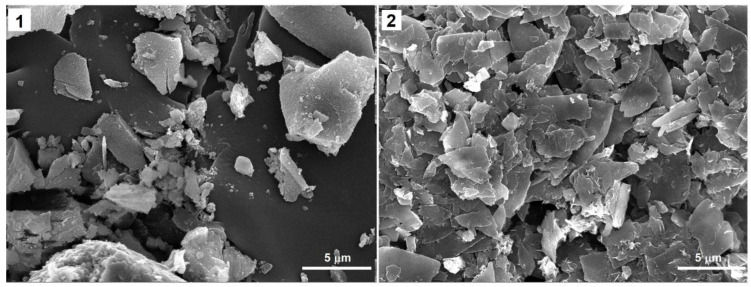
FE-SEM images of **1** and **2**.

FE-SEM (field-emission scanning electron microscopy) images of both the compounds were observed to examine the morphologies as well as the self-assembly behaviors. To prepare the samples, both compounds were suspended in a 1:1 solvent mixture of water and acetone (C = 1.5 mM), followed by centrifugation at a speed of 13,500 rpm for 20 s, and then drop-casted on the surface of copper tapes. Then, the sample foil was dried in air. Pt coating was necessary before the FE-SEM studies. The images of the self-assembled nanoaggregates of **1** and **2** are given in Figure 10. It is clear from the FE-SEM images that the morphologies of **1** and **2** are significantly different. Compound **1** comprises nanostructures without specific dimensions. However, **2** exhibits a high-density nanoplate morphology with smooth surfaces. The ionic porphyrin-based nanoplates were irregularly shaped, where the average size and thickness of the smaller plates were 1200 × 600 nm and ≈50 nm, respectively. The average diameter of the bigger nanoplates differed from 3500 × 1500 nm, and their thickness was ≈50 nm.

### 2.3. Catalytic Photodegradation of Pollutants

**Figure 11:**
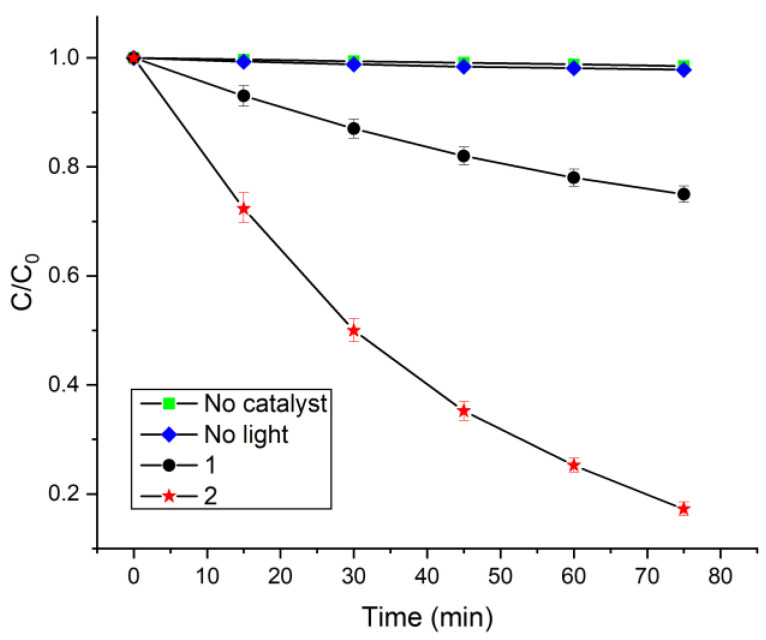
Catalytic photodecomposition of MO dye in water (pH = 6.3 and temperature = 298 K).

The photocatalytic activities of 1 and 2 were analyzed through the visible-light photodegradation of the aqueous solution of toxic contaminants. MO dye and TC were chosen as model contaminants for the catalytic degradation experiments. The time-dependent optical absorption spectra of MO dye in the presence of complex 2 are given in Figure S6. Without a visible light or catalyst, MO slightly decomposed (Figure 11). Therefore, catalysts as well as light are necessary for the photocatalytic photodegradation of MO dye. The photocatalytic degradation of MO was analyzed by determining the absorbance band at 462 nm with increasing irradiation time. The catalytic performances of both compounds in MO dye degradation were determined by calculating the decomposition efficiency, (*C*_0_ − *C*)/*C*_0_, where *C*_0_ is the initial concentration of MO dye and C is the concentration at time *t*. Figure 11 clearly shows that **2** is more efficient at photocatalytically degrading MO dye than **1**, decomposing MO completely within 75 min. The photocatalytic photodegradation efficiencies of **2** and **1** reached 83% and 30%, respectively. The kinetics of MO dye decay were modeled using a pseudo-first-order rate equation, represented by ln(*C*_0_/*C*) = *k*t, which is generally employed for catalytic decomposition reactions if the initial concentration of the model pollutant is low. In the equation, *k* is the pseudo-first-order decay rate constant. The photodegradation rate of MO dye (Figure S7) was obtained from the plot of ln(*C*_0_/*C*) vs. *t* in Figure 11. The pseudo-first-order photodecomposition rate constant for MO by **2** (0.023 min^−1^) is ≈4.6 times better than that of **1** (0.005 min^−1^). These rate constants are more promising than those obtained by other catalysts used to degrade MO dye (Table 1).

**Figure 12:**
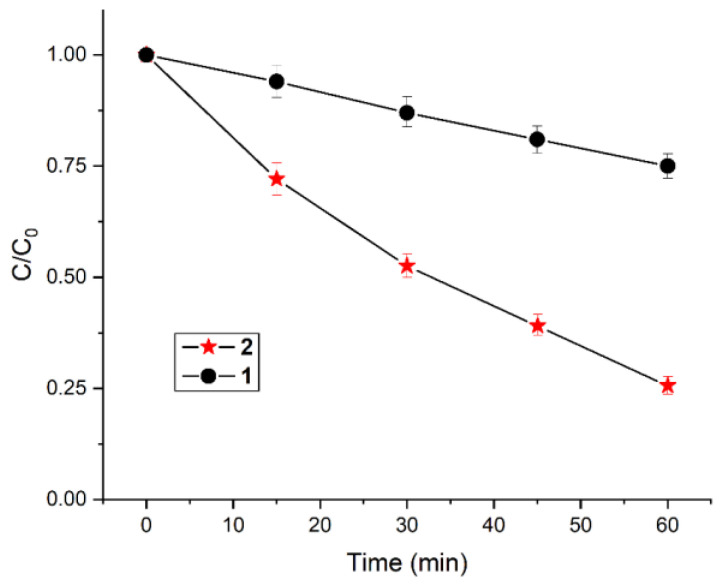
Visible-light degradation of TC antibiotic in water (pH = 5.8, temperature = 298 K).

The universality of the catalytic photodegradation efficiencies of 1 and 2 was further demonstrated in the degradation of the TC antibiotic. The time-dependent absorption spectra for the photodegradation of TC in the presence of catalyst **2** are depicted in Figure S8. The photocatalytic decay of TC was analyzed by computing the absorbance peak at 356 nm with increasing irradiation time. Within 60 min of visible-light irradiation, the photodegradation ratios of the photocatalysts derived from **2** and **1** reached 75% and 25%, respectively (Figure 12). The decomposition rate constant (pseudo-first-order) for the degradation of TC by 1 (0.004 min^−1^) is lower than that of 2 (0.018 min^−1^) (Figure S9). Table 2 compares the catalytic performances attained in this work with those of other catalysts used for TC degradation.

From the above data, it is evident that **1** and **2** exhibited excellent catalytic degradation of pollutants in water. The reusability of the catalyst is vital for industrial purposes, which was assessed by performing recycling experiments of **2** following MO dye degradation. As displayed in Figure S10, **2** maintains its high catalytic MO degradation performance, with a slight decrease (≈10%) after five continuous cycles (375 min). The degradation performance of **2** gradually decreased with the number of times it was recycled, which might be due to byproducts covering the surface of photocatalyst **2**. The process of photocatalyst recovery after the degradation experiments was not straightforward due to the high solubility of **1** and **2** in water. After the fifth cycle, acetone was added to the reaction mixture with constant stirring. The mixture was filtered, and the precipitate was rinsed with acetone and then dried. The morphology of spent catalyst **2** was studied using FE-SEM. The images in Figure S11 confirm that the morphologies of the spent catalyst and fresh catalyst did not differ significantly. Additionally, the FTIR spectra (Figure S12) and PXRD spectra (Figure S13) of **2** indicate the robustness of the catalyst during the photodegradation experiment. This confirms that visible degradation efficiency and stability of the catalyst remained unaffected, which is promising for pollutant degradation.

The optimum conditions for the photodegradation experiments were also determined, including the temperature, ratio of MO dye to catalyst, and pH of the solution. For this motive, MO was degraded at varying temperatures to observe the temperature effect on the MO dye removal. We found that the MO degradation efficiency of the catalyst increases gradually with temperature (Figure S14), where the pH of the MO dye solution affects the photodegradation rate (Figure S15). Figure S15 indicates that the removal rate of MO dye remains unchanged within the acidic pH range, but the rate drastically decreases as the pH of the solution increases. This implies that catalyst **2** lost its degradation activity once deprotonated in a strongly basic medium and seemed to behave like **1** in basic media. To ascertain the effect of the MO dye/photocatalyst ratio on the photocatalytic degradation reaction, we varied the concentration of the MO dye (10–60 mg L^−1^) while keeping the concentration of catalyst **2** constant (30 mg/L). It was noticed that the photocatalytic removal rate of MO dye decreases as the dye concentration increases (Figure S16). Higher dye concentrations are more likely to prevent visible light from reaching the catalyst surface. Furthermore, to examine the influence of light intensity on the MO dye photodegradation rate using **2**, we applied monochromatic light of varying wavelengths (Figure S17). A significant variation in the rate was observed; this is likely because the wavelength of the light has a significant influence on solar energy harvesting and photodecomposition activity. Furthermore, **2** still showed slight photocatalytic decomposition ability, even at λ > 650 nm.

ESI-MS was used to determine the characteristics of MO dye degradation residues in the presence of **2** (Figure S18)**.** For this, **1** mL of reaction mixture was taken from the vessel after 30 min and analyzed. The new peaks in the ESI-MS confirm the decomposition of MO into different fragments [73]. Based on Figure S18, possible intermediates for the decay of MO dye are depicted in Figure S19. At the beginning, the base peak (*m*/*z* = 304.1) is associated with the anionic form of MO. MO then underwent two successive *N*-demethylation reactions and disintegrated into an intermediate with an *m*/*z* of 276.0. This anionic fragment ruptured further after the cleavage of the azo bond into low-molecular-weight residues with *m*/*z* values of 172.1 and 135.0. These fragments further underwent sequential ring opening and hydrolysis, producing smaller fragments with *m*/*z* values of 109.0 and 99.0. In the end, all the intermediates had disintegrated into small molecules, finally mineralizing into H_2_O and less toxic CO_2_. Moreover, the TOC (total organic carbon) removal efficiency of **2** (78%) was estimated to exceed that of **1** (49%) for the photodecomposition of MO dye [52].

### 2.4. Possible Mechanism of the Catalytic Photodegradation of Pollutants

In the experiments described so far, **2** has shown a higher photocatalytic activity for pollutant degradation than **1**. Photodegradation performance likely depends on some key factors such as specific surface area, bandgap energy (E_g_), the surface morphology of the catalyst, and light-harvesting capacities. Photogenerated charge separation, carrier transportation, and reactive species lifetime also play important roles in the degradation reaction. E_g_ was estimated from the Tauc plot derived from the absorption spectra in Nujol (Figures S4 and S20). The E_g_ of **2** is ≈2.58 eV, which is lower than that of **1** (≈2.94 eV); therefore, **2** has better light-harvesting capacity than **1**. As shown in Figure S21 (λ_ext_ = 550 nm), the fluorescence intensity of **2** (≈35%) is markedly lower than that of **1**, indicating that reunification of photogenerated reactive pairs is restricted and strong electronic delocalization occurs over the surface of the conjugated aromatic skeletons.

Photoelectrochemical experiments were conducted to check the role of the charge transfer complex in the catalytic decomposition reactions of **1** and **2**. As depicted in Figure S22, **2** displays a 2.6-fold higher photocurrent response than **1**, demonstrating its superior charge carrier separation. Moreover, under visible-light exposure, the electrochemical impedance spectra (Nyquist plot) of **2** exhibit a lower arc radius than those of **1** (Figure S23), signifying that the working electrode has a lower charge transfer resistance [74]. Therefore, **2** is superior to **1** in terms of the separation and transportation of photogenerated reactive pairs.

Radical trapping tests were examined to elucidate the production of the photogenerated reactive species during the catalytic degradation experiment [75,76,77]. For this investigation, sodium azide (NaN_3_) was utilized to trap singlet O_2_, *tert*-butanol (^t^BuOH) was utilized to seize the hydroxyl radical (^•^OH), ethylenediaminetetraacetic acid disodium (Na_2_-EDTA) was applied to seize photogenerated holes (h^+^), and *para*-benzoquinone (*p*-BQ) was employed to capture superoxide radical anion (O_2_^−•^) during the photodegradation of MO dye in the presence of **2** (Figure S24). As depicted in Figure S24, the photodegradation performance of **2** was seriously influenced by the presence of *p*-BQ, Na_2_-EDTA, and ^t^BuOH. However, the decomposition of MO dye was not influenced by NaN_3_ (i.e., singlet oxygen). The photocatalytic decomposition rate of MO dye decreased remarkably in the presence of Na_2_-EDTA compared with either ^t^BuOH or *p*-BQ. These results clearly show that photogenerated holes (h^+^) are the dominant reactive species that influence the catalytic decomposition ability of **2**.

Based on the above investigation, the mechanism of charge transfer, carrier separation, and degradation was proposed for the porphyrin-based catalyst (P_cat_). Upon exposure to visible light, P_cat_ is excited after surpassing the bandgap energy, generating a significant number of electron–hole pairs (e^−^/h^+^) in the process. Afterward, the excited electrons prompt high electronic delocalization over the surfaces of conjugated aromatic frameworks in P_cat_ and delay the reunification time. This assists the creation of abundant photogenerated charge pairs and facilitates their transportation and separation from association. Hydroxyl radical (^•^OH) is produced in the reaction of H_2_O with a photogenerated hole (h^+^) and decomposes the MO dye into small fragments. Superoxide radical anion (O_2_^−•^) is generated in the reaction of O_2_ with excited electrons and decomposes the MO dye into small fragments. Lastly, all the low-molecular fragments mineralize, forming H_2_O and less toxic CO_2_. In general, the mechanism for a porphyrin-containing catalyst (P_cat_) consists of five steps:

P_cat_ + *hv* **→** P_cat_ ^∗^ (e^−^ + h^+^) (1)

H_2_O + h^+^**→**^•^OH + H^+^ (2)

O_2_ + e^−^**→**O_2_^−•^ (3)

MO + O_2_^−•^**→**Decomposed products (4)

MO + ^•^OH**→**Decomposed products (5)

Porphyrin **2** showed much better catalytic degradation activity than **1**, which could be for the following reasons: (i) Porphyrin **2** has a remarkably stronger light absorption capacity than **1**, thus generating more photogenerated reactive species that facilitate the catalytic degradation reaction. (ii) The assembled structure of **2** can highly stabilize the photogenerated reactive pairs over the aromatic conjugated frameworks and deliver multidimensional channels to transfer charge carriers, thus delaying the recombination time. (iii) The permanent porosity of **2**, combined with its active specific surface area, facilitates the adsorption of pollutants, again enhancing the degradation reaction. (iv) Multidimensional interactions between **2** and the pollutants also increase the degradation rate. (v) Excess reactive species (h^+^, ^•^OH, and O_2_^−•^) are created in the photodegradation reaction catalyzed by **2** compared with that catalyzed by **1**.

## 3. Materials and Methods

All starting compounds were procured from TCI (Tokyo Chemical Industry Co., Ltd., Tokyo, Japan) and applied without further purification unless otherwise mentioned. [Sn(OH)_2_(TPyP)] (**1**) or *trans*-dihydroxo[5,10,15,20-tetrakis(4-pyridyl)porphyrinato]tin(IV) was synthesized using our previously reported method [72]. Elemental analyses were carried out using a ThermoQuest EA 1110 analyzer (Thermo Fisher Scientific, Waltham, MA, USA). A UV-3600 spectrophotometer (Shimadzu, Tokyo, Japan) was used to measure UV–vis absorption spectra. A Shimadzu RF-5301PC fluorescence spectrophotometer was used to measure fluorescence spectra. FTIR spectra (KBr pellet) were obtained using a Shimadzu FTIR-8400S spectrophotometer (Shimadzu, Tokyo, Japan). PXRD patterns were measured using an AXS D8 Advance powder X-ray diffractometer (Bruker, Billerica, MA, USA). TGA was obtained using an Auto-TGA Q500 instrument (TA Instruments, New Castle, DE, USA). A MAIA-III field-emission scanning electron microscope (TESCAN, Brno, Czech Republic) was used to capture FE-SEM images. BET surface areas were determined using a BELSORP-mini volumetric gas adsorption analyzer (ATS Scientific, Burlington, ON, Canada) using N_2_ adsorption isotherms at 77 K.

### 3.1. Synthesis of [Sn(H_2_PO_4_)_2_(TPy^H^P)](H_2_PO_4_)_4_∙6H_2_O (**2**)

In a typical procedure, 154.0 mg (0.20 mmol) of [Sn(OH)_2_(TPyP)] (**1**) was dissolved in 3 mL of 1% phosphoric acid (H_3_PO_4_) aqueous solution and poured into a 40 mL long tube. Afterward, acetone (30 mL) was added to facilitate crystal growth by slow diffusion and allowed to stand in the dark. After 7 days, block-shaped red violet crystals of **2** were collected from the bottom of the tube. The dark violet crystalline materials were filtered, rinsed with excess acetone, and then dried in air. Yield: 223 mg (90%). ^1^H NMR (400 MHz, DMSO-d_6_): *δ* 9.57 (s, 8H, H-pyrrole), 9.41 (d, *J* = 6.4 Hz, 8H, H2,6-Py), 9.12 (d, *J* = 6.2 Hz, 8H, H3,5-pyridine). FTIR (ν/cm^−1^, KBr pellet): 3100 (w), 1630 (s), 1495 (s), 1345 (w), 1250 (br), 1220 (br), 1090 (w), 1030 (w), 955 (vs), 845 (w), 785 (s), 710 (w), 552 (w), and 545 (w). UV–vis (DMSO, nm): *λ*_max_ (log ε) 416 (4.60), 512 (2.51), 550 (3.24), and 590 (2.62). UV–vis (Nujol, nm): λ_max_ 432, 520, 572, and 610. Emission (DMSO, nm): *λ*_nm_ 610 and 664. Emission (Nujol, nm): *λ*_nm_ 644 (br). Elemental analysis after the removal of the solvents: anal. calcd. for C_40_H_40_N_8_O_24_P_6_Sn (%): C, 36.36; H, 3.05; N, 8.48; and R, 52.11. Found: C, 36.18; H, 3.21; N, 8.39; and R, 52.22. ESI-MS: *m*/*z* 233.67 for [Sn(H_2_PO_4_)_2_TPy*^H^*P]^4+^ (required: 233.52) and *m*/*z* 129.39 for [Sn(OH_2_)_2_TPy*^H^*P]^6+^ (required: 129.31).

### 3.2. X-ray Single-Crystal Structure Analysis

A crystal of **2** appropriate for single X-ray diffraction experiments was obtained from the bottom of the tube. After that, it was immersed in Paratone-N hydrocarbon oil on glass fiber to avoid the loss of solvent molecules and then positioned on a Bruker APEX-II CCD diffractometer (Bruker, Billerica, MA, USA) equipped with a charge-coupled device (CCD) detector. A graphite monochromated Mo Kα (*l* = 0.71073 Å) was used as the radiation source. The data were collected at 130 K under N_2_ flow. The structure was solved using direct methods with the SHELXTL program (Version 6.10) [78,79]. Full-matrix least-squares methods were used to refine the data. The graphical works were obtained by using the Olex2 software package (version 1.5) [80]. Anisotropic thermal parameters were used to refine the non-hydrogen atoms. The hydrogen atoms bonded to nitrogen and carbon were incorporated in geometric positions and prescribed thermal parameters equivalent to 1.2 times those of the atom to which they were bonded.

### 3.3. Photoelectrochemical Experiment

The electrochemical impedance and photocurrent response were examined using an electrochemical work station (CHI660D) and 0.1 M aqueous solution of Na_2_SO_4_ as the electrolyte. The 3-electrode system consisted of a saturated calomel electrode, a Pt wire electrode, and a working electrode. The working electrode was developed as follows: **1** and **2** (~6 mg) were dispersed in 5.0 mL of ethanol by sonication, followed by deposition on the surface of indium tin oxide. After that, the surface was dried in air, followed by heating at 170 °C for 12 h. A 150 W xenon arc lamp was used as a visible-light source in the photoelectrochemical experiments.

### 3.4. Catalytic Photodegradation Measurement

The catalytic activities of **1** and **2** were examined by the photocatalytic decomposition of the MO dye and TC antibiotic in distilled water. A 150 W xenon arc lamp (ABET Technologies, Milford, CT, USA) was selected as the source of visible light with a UV cut-off filter. An amount of 15 mg of **1** or **2** was added to 500 mL of water containing MO dye (30 mg L^−1^) and constantly stirred. The solution was kept in the dark for 30 min to establish an adsorption–desorption equilibrium. Then, 3 mL of the solution was taken out after light irradiation at regular intervals. The accurate concentration of MO was calculated by determining the absorbance at 462 nm using UV–vis spectroscopy. TC solution was prepared from tetracycline hydrochloride in water (pH = 6.0, 125 mg L^−1^). The accurate concentration of TC was estimated by calculating the absorbance at 356 nm using UV–vis spectroscopy (Shimadzu, Tokyo, Japan).

## 4. Conclusions

[Sn(H_2_PO_4_)_2_(TPy*^H^*P)](H_2_PO_4_)_4_∙6H_2_O (**2**) was synthesized from the reaction of [Sn(OH)_2_TPyP] (**1**) with H_3_PO_4_. The single X-ray crystal structure of complex **2** disclosed that the ionic complex [Sn(H_2_PO_4_)_2_TPy*^H^*P]^4+^ comprises four peripheral pyridinium moieties and two anionic axial Sn–O–PO(OH)_2_ units. The overall tetra-cationic charge was balanced by four dihydrogen phosphate (H_2_PO_4_^−^) anions. Intermolecular hydrogen bonds between the protonated pyridylporphyrins and axially coordinated phosphate ions gave rise to the formation of a network architecture with a porous channel occupied by the anions and water molecules. The construction of **2** from the reaction of **1** with H_3_PO_4_ not only alters the structural architecture but also modifies the structure to impart a high surface area, high thermodynamic stability, interesting surface morphology, and excellent catalytic photodegradation of water contaminants. The photodegradation efficiency of **2** toward MO dye was observed to be 83% within 75 min at a rate constant of 0.023 min^−1^, while its efficiency for the degradation of TC was 75% within 60 min at a rate constant of 0.018 min^−1^. This work represents progress in the advancement of highly efficient visible-light porphyrin-containing ionic self-assembled catalytic systems. It also highlights the application of such photocatalysts in wastewater treatment.

## Figures and Tables

**Table 1:** Description of the photocatalytic degradation efficiency of MO with several visible-light catalysts.

Photocatalysts	Irradiation Time (min)	Catalyst Dosage (mg/L)	Dye Concentration (mg/L)	Rate Constant (min^−1^)	Reference
BiOI–BiOBr	300	2000	10	0.0031	[41]
BiOBr	80	1000	10	0.0072	[42]
15% TiO_2_/BiOBr	80	1000	10	0.0243	[42]
graphene@porphyrin (GNPs@TCPP)	180	5	5	0.0065	[43]
*g*-C_3_N_4_	120	1000	10	0.0038	[44]
WO_3_	120	1000	10	0.0018	[44]
WO_3_-*g*-C_3_N_4_	120	1000	10	0.0213	[44]
Zn(II)-porphyrin/poly(acrylic acid)	180	50	10	0.048	[45]
Bi_2_MoO_6_–Bi_5_O_7_Br	180	200	16	0.0035	[46]
Ag_2_Mo_1–*x*_W*_x_*O_4_ (x = 0.50)	140	1000	5	0.0054	[47]
Zn_0.999_Gd_0.001_O	90	400	15	0.0212	[48]
Mn_3_O_4_/ZnO/Eu_2_O_3_	150	150	5	0.0107	[49]
TiO_2_	80	500	20	0.015	[50]
5.0 wt% MoS_2_-TiO_2_	80	500	20	0.028	[50]
ZnO	120	1000	100	0.005	[51]
10% Co–ZnO	120	1000	100	0.014	[51]
Zn(II)–Sn(IV)–Zn(II) Porphyrin-Triad	100	67	20	0.0251	[52]
4α-[Zn(TAzPP)]	70	200	20	0.016	[53]
[H4TTP]Cl_2_∙3CHCl_3_ + H_2_O_2_	180	500	30	0.0042	[54]
Ni(TAMPP) + H_2_O_2_	90	500	30	0.013	[55]
**1**	75	30	30	0.005	This work
**2**	75	30	30	0.023	This work

**Table 2:** Comparison of TC decomposition performances of different photocatalysts.

Photocatalyst	Irradiation Time (min)	Catalyst Dose (mg/L)	TC Concentration (mg/L)	Rate Constant (min^−1^)	Reference
AgIn(MoO_4_)_2_	60	1000	10	0.00698	[56]
6%-Ag/AgIn(MoO_4_)_2_	60	1000	10	0.00985	[56]
Ag_2_CO_3_/Ag/WO_3_	90	1000	10	0.0179	[57]
polyacrylonitrile (PAN)-TiO_2_/Ag	240	1000	20	0.013	[58]
g-C_3_N_4_	180	1000	10	0.0032	[59]
CdIn_2_S_4_	180	1000	10	0.00422	[59]
rGO/g-C_3_N_4_	180	1000	10	0.00251	[59]
rGO/CdIn_2_S_4_	180	1000	10	0.0042	[59]
CdIn_2_S_4_/g-C_3_N_4_	180	1000	10	0.0043	[59]
rGO/30%- CdIn_2_S_4_/g-C_3_N_4_	180	1000	10	0.00766	[59]
CuBi_2_O_4_	120	500	10	0.00341	[60]
Fe_2_O_3_	120	500	10	0.00485	[60]
30%- Fe_2_O_3_/CuBi_2_O_4_	120	500	10	0.01246	[60]
CuBi_2_O_4_	120	500	10	0.0032	[61]
MoS_2_	120	500	10	0.0034	[61]
CuBi_2_O_4_/MoS_2_ (5%)	120	500	10	0.0095	[61]
La_2-x_Sr_x_NiMnO_6_ (x = 0.10)	240	1000	10	0.0101	[62]
CdS	90	250	40	0.0056	[63]
γ-In_2_Se_3_ (0.04 M EDTA)	120	1000	20	0.0175	[64]
TiO_2_ (P-25)	150	200	10	0.0069	[65]
BP (black phosphorus)	90	1000	50	0.0007	[66]
BiOBr	90	1000	50	0.0027	[66]
10BP/BiOBr	90	1000	50	0.0110	[66]
AgFeO_2_	60	500	40	0.0060	[67]
Ag/AgFeO_2_	60	500	40	0.0104	[67]
poly vinylidene fluoride-TiO_2_@g-C_3_N_4_-0.2g	300	1000	50	0.0120	[68]
BiVO_4_	240	250	10	0.0045	[69]
Bi_2_WO_6_	180	300	20	0.0044	[70]
Oxygen deficient Bi_2_WO_6_	180	300	20	0.0078	[70]
Zn(II)-Sn(IV)-Zn(II) porphyrin triad	45	50	100	0.0260	[71]
**1**	60	30	125	0.0040	This work
**2**	60	30	125	0.0180	This work

## Data Availability

Crystallographic data have been deposited at the Cambridge Crystallographic Data Center under the reference number 2377416.

## Supplementary Materials

The following supporting information can be downloaded at: https://www.mdpi.com/article/10.3390/molecules29174200/s1: Table S1. Crystallographic data and structural refinements for 2. Table S2. Selected bond lengths [Å] and angles [°] for 2. Figure S1. ^1^H NMR spectrum of 2 in DMSO-d_6_. Figure S2. ESI mass spectrum of 2. Figure S3. Adsorption and desorption isotherms of N_2_ for 1 and 2 at 77 K. Figure S4. UV–vis spectra of 1 and 2 in Nujol (10 mg/mL). Figure S5. TGA thermogram of 2. Figure S6. Absorption spectra of MO dye in the presence of 2 under visible-light irradiation. Figure S7. Kinetics of the photocatalytic degradation of MO under visible-light irradiation. Figure S8. Absorption spectra of TC in the presence of 2 under visible-light irradiation. Figure S9. Kinetics of the photocatalytic degradation of TC under visible-light irradiation. Figure S10. Recyclability of photocatalyst 2 toward the degradation of MO dye. Figure S11. FE-SEM images of 2 (after and before the degradation of MO). Figure S12. FTIR spectra of 2 (after and before the degradation of MO dye). Figure S13. PXRD spectra of 2 (after and before the degradation of MO dye). Figure S14. Effect of temperature on the photocatalytic degradation of MO dye in the presence of 2. Figure S15. Effect of pH on the degradation of MO dye solution in the presence of 2. Figure S16. Effect of MO dye concentration on the photocatalytic degradation of MO in the presence of 2. Figure S17. Effect of light intensity on the photocatalytic degradation of MO dye in the presence of 2. Figure S18. Negative-ion-mode ESI mass spectrum of MO dye degradation catalyzed by 2 after 30 min of visible-light irradiation. Figure S19. Possible intermediates of the MO dye degradation reaction in the presence of 2 after 30 min of visible-light irradiation. Figure S20. Bandgap energies of 1 and 2 were calculated from the Tauc plots using absorption spectroscopy data. Figure S21. Fluorescence spectra of 1 and 2 in Nujol (C = 10 mg/mL). λ_ex_ = 550 nm. Figure S22. Photocurrent responses for 1 and 2 under visible light. Figure S23. EIS–Nyquist plots of 1 and 2 under visible light. Figure S24. Visible-light MO dye degradation activities of 2 in the presence of various scavengers.
