# Supplementary material for: Self-Assembled Nanostructure of Ionic Sn(IV)porphyrin Complex Based on Multivalent Interactions for Photocatalytic Degradation of Water Contaminants
Source: Molecules. 2024 Sep 4;29(17):4200. doi: 10.3390/molecules29174200 (PMC11539948; doi:10.3390/molecules29174200)
Supplement: Supplementary file 1 [file molecules-29-04200-s001.zip › molecules-3182266-supplementary.pdf]

Supplementary Materials

Self-Assembled Nanostructure of Ionic Sn(IV)porphyrin Complex Based on Multivalent Interactions for Photocatalytic Degradation of Water Contaminants

Nirmal Kumar Shee and Hee-Joon Kim *

Department of Chemistry and Bioscience, Kumoh National Institute of Technology, Gumi 39177, Republic of Korea; nirmalshee@gmail.com

* *Correspondence: hjk@kumoh.ac.kr; Tel.: +82-54-4787822*

List of contents:

Table S1. Crystallographic data and structural refinements for **2**.

Table S2. Selected bond lengths [Å] and angles [°] for **2**.

Figure S1. ^{1}H NMR spectrum of **2** in DMSO-d_6.

Figure S2. ESI mass spectrum of **2**.

Figure S3. Adsorption and desorption isotherms of N_2 for **1** and **2** at 77 K.

Figure S4. UV–vis spectra of **1** and **2** in Nujol (10 mg/mL).

Figure S5. TGA thermogram of **2**.

Figure S6. Absorption spectra of MO dye in the presence of **2** under visible-light irradiation.

Figure S7. Kinetics of the photocatalytic degradation of MO under visible-light irradiation.

Figure S8. Absorption spectra of TC in the presence of **2** under visible-light irradiation.

Figure S9. Kinetics of the photocatalytic degradation of TC under visible-light irradiation.

Figure S10. Recyclability of photocatalyst **2** toward the degradation of MO dye.

Figure S11. FE-SEM images of **2** (before and after the degradation of MO).

Figure S12. FTIR spectra of **2** (before and after the degradation of MO dye).

Figure S13. PXRD spectra of **2** (before and after the degradation of MO dye).

Figure S14. Effect of temperature on the photocatalytic degradation of MO dye in the presence of **2**.

Figure S15. Effect of pH on the degradation of MO dye solution in the presence of **2**.

Figure S16. Effect of MO dye concentration on the photocatalytic degradation of MO in the presence of **2**.

Figure S17. Effect of light intensity on the photocatalytic degradation of MO dye in the presence of **2**.

Figure S18. Negative-ion-mode ESI mass spectrum of MO dye degradation catalyzed by **2** after 30 min of visible-light irradiation.

Figure S19. Possible intermediates of the MO dye degradation reaction in the presence of **2** after 30 min of visible-light irradiation.

Figure S20. Bandgap energies of **1** and **2** were calculated from the Tauc plots using absorption spectroscopy data.

Figure S21. Fluorescence spectra of **1** and **2** in Nujol (C = 10 mg/mL). λ_{ex} = 550 nm.

Figure S22. Photocurrent responses for **1** and **2** under visible light.

Figure S23. EIS–Nyquist plots of **1** and **2** under visible light.

Figure S24. Visible-light MO-dye degradation activities of **2** in the presence of various scavengers.

Table S1. Crystallographic data and structural refinements for **2**.

	**2**
Empirical formula	$C_{40}H_{52}N_8O_{30}P_6Sn$
Formula weight	1429.43
Crystal size (mm^3)	0.2 × 0.15 × 0.08
T (K)	130
Crystal system, Space group	Monoclinic, P 1 2_1/c 1
Cell dimensions	
a (Å)	12.8867(2)
b (Å)	9.8090(1)
c (Å)	21.3784(3)
α (deg)	90.00
β (deg)	91.786(1)
γ (deg)	90.00
V (Å^3)	2701.04(6)
Z, D_c (g cm^{-3})	4, 1.743
μ (mm^{-1})	0.754
F(000)	1433.129
θ range (°)	1.58 to 28.30
Reflections collected	25,592
Independent reflections (R_{int})	6650
Absorption correction	None
Data / restraints / parameters	6650 / 0 / 452
GOF on F^2	1.0399
$R1$,[a] $wR2$[b] [$I>2\sigma(I)$]	0.0284, 0.0733
$R1$,[a] $wR2$[b] (all data)	0.0320, 0.0757
Largest peak/hole (e Å^{-3})	1.0874/−1.0014

[a] $R1 = \Sigma\|F_o| - |F_c\|/\Sigma|F_o|$. [b] $wR2 = [\Sigma[w(F_o^2 - F_c^2)^2]/\Sigma[w(F_o^2)^2]]^{1/2}$.

Table S2. Selected bond lengths [Å] and angles [°] for **2**.

Sn1−N1 2.0837(15)	Sn1−N2 2.0800(15)	Sn1−O1 2.0838(13)	P1−O1 1.5419(13)
P1−O2 1.5377(15)	P1−O3 1.5627(14)	P1−O4 1.5101(14)	P2−O5 1.5633(16)
P2−O6 1.5166(15)	P2−O7 1.5606(16)	P2−O8 1.4988(15)	P3−O9 1.5656(16)
P3−O10 1.5096(16)	P3−O11 1.5013(15)	P3−O12 1.5669(17)	

N1−Sn1−N1 180.0	N2−Sn1−N1 90.26(6)	N2−Sn1−N1 89.74(6)
N2−Sn1−N2 180.0	O1−Sn1−N1 92.62(6)	O1−Sn1−N1 87.38(6)
O1−Sn1−N2 90.13(6)	O1−Sn1−N2 89.87(6)	O1−Sn1−O1 180.0
O2−P1−O1 111.75(8)	O3−P1−O1 105.98(8)	O3−P1−O2 105.41(8)
O4−P1−O1 109.30(8)	O4−P1−O2 113.20(8)	O4−P1−O3 110.91(8)
P1−O1−Sn1 134.94(8)	O6−P2−O5 108.63(9)	O7−P2−O5 101.14(9)
O7−P2−O6 110.82(9)	O8−P2−O5 111.90(10)	O8−P2−O6 113.42(9)
O8−P2−O7 110.26(9)	O10−P3−O9 111.46(9)	O11−P3−O9 108.82(9)
O11−P3−O10 112.45(9)	O12−P3−O9 102.00(9)	O12−P3−O10 110.45(10)
O12−P3−O11 111.18(10)		

Symmetry codes: (i) x, y, z; (ii) -x, 1/2+y, 1/2-z; (iii) -x, -y, -z; (iv) x, 1/2-y, 1/2+z.

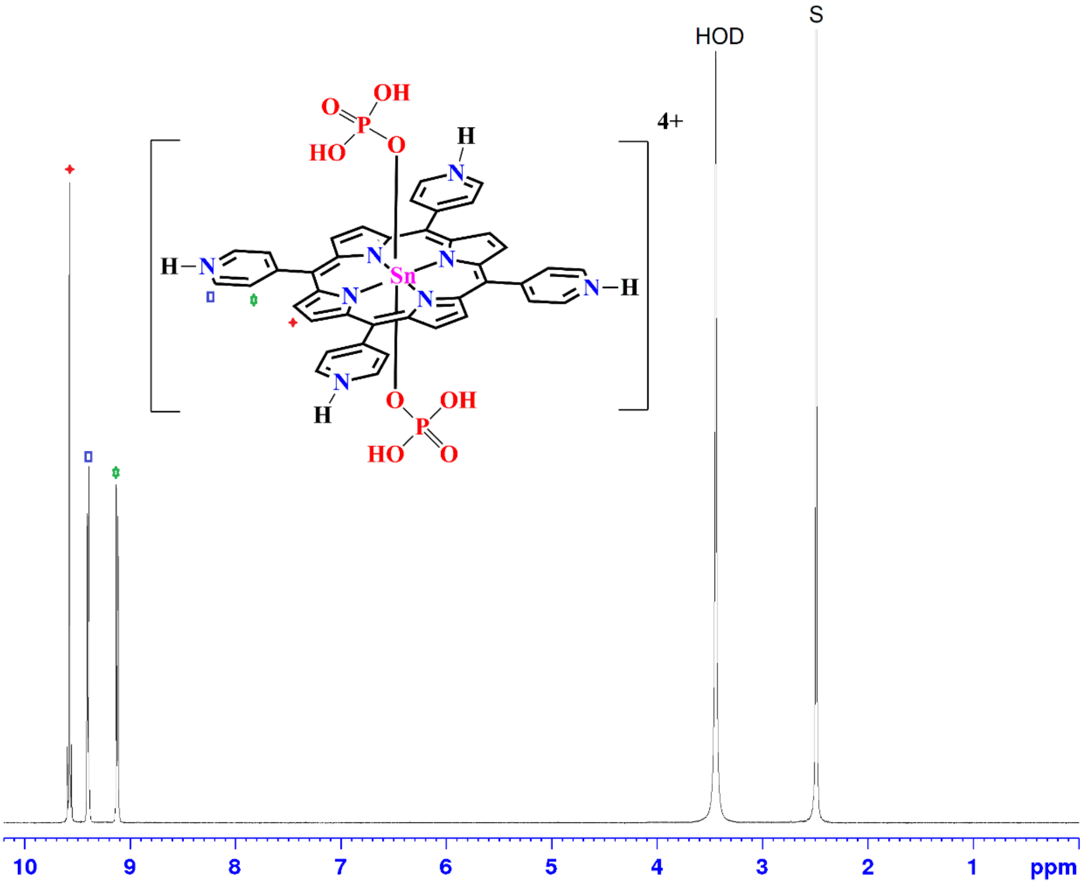

Figure S1. ^{1}H NMR spectrum of **2** in DMSO-d$_6$.

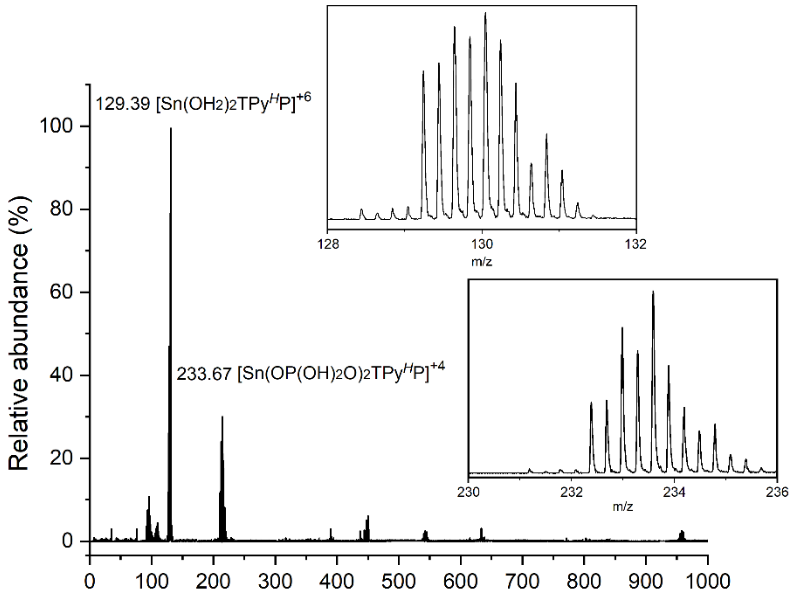

Figure S2. ESI mass spectrum of **2**.

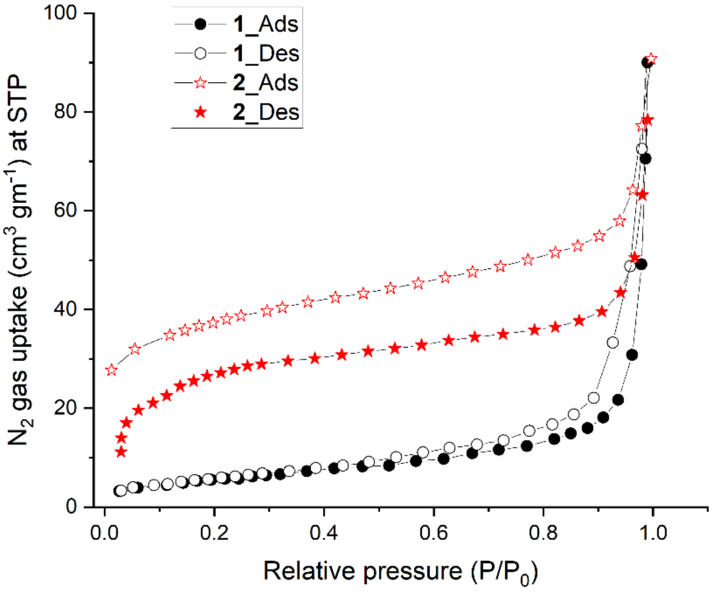

Figure S3. Adsorption and desorption isotherms of N_2 for **1** and **2** at 77 K.

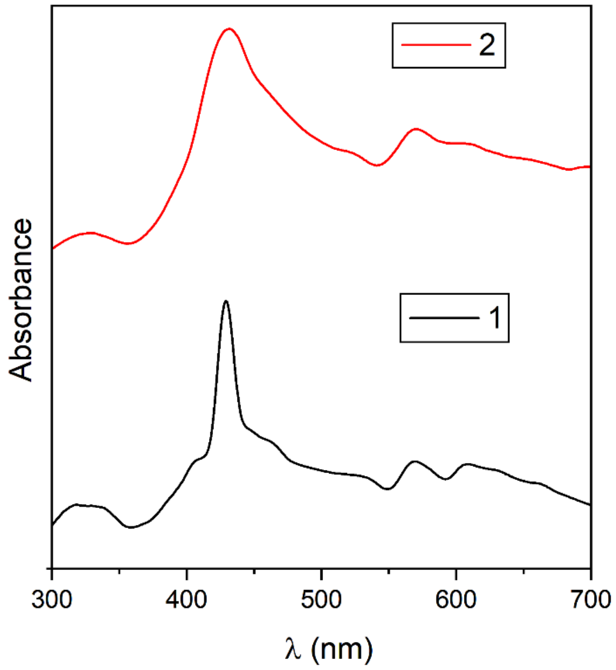

Figure S4. UV–vis spectra of **1** and **2** in Nujol (10 mg/mL).

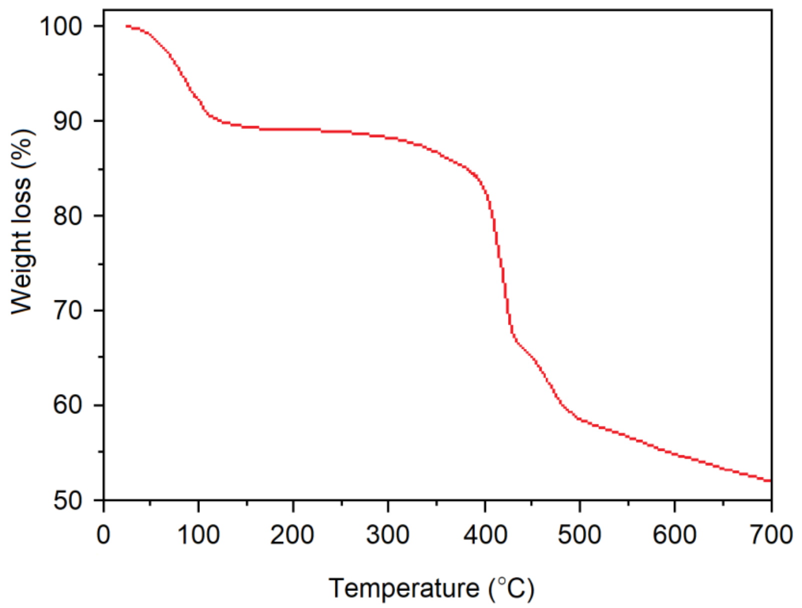

Figure S5. TGA thermogram of **2**.

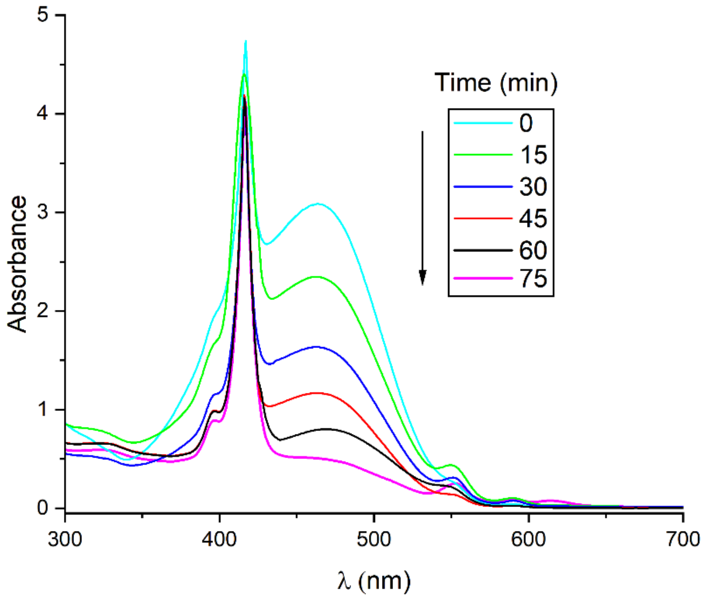

Figure S6. Absorption spectra of MO dye in the presence of **2** under visible-light irradiation.

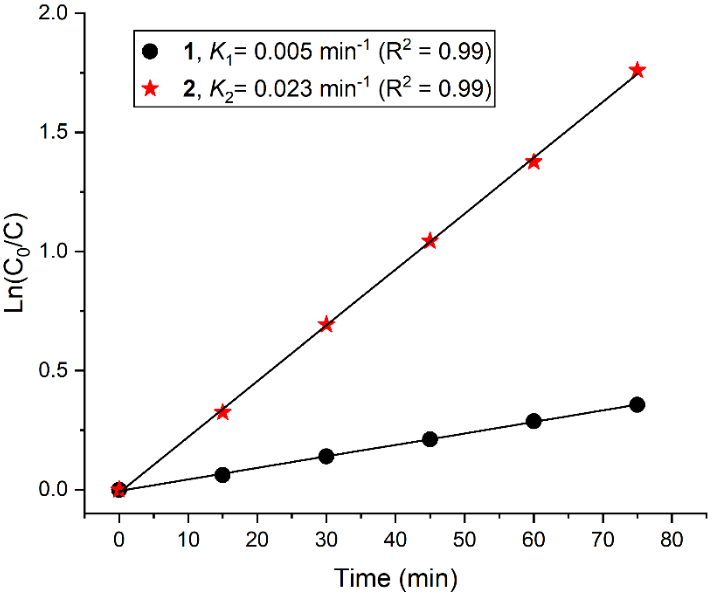

Figure S7. Kinetics of the photocatalytic degradation of MO under visible-light irradiation.

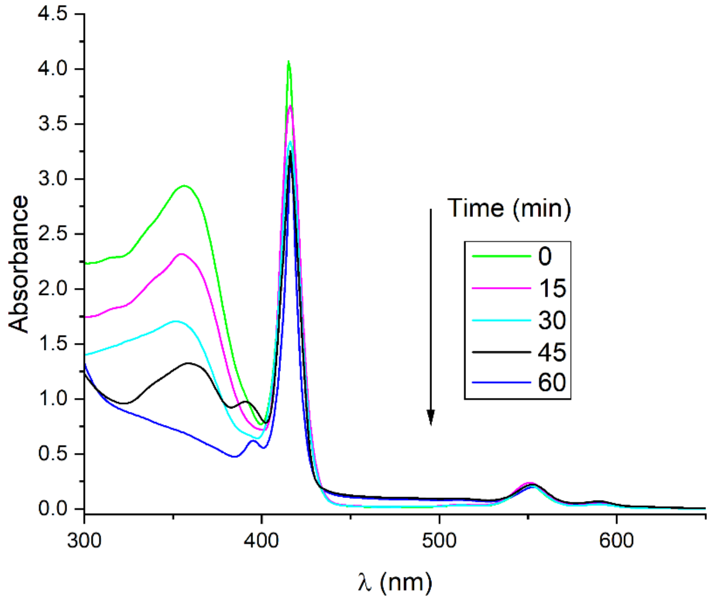

Figure S8. Absorption spectra of TC in the presence of **2** under visible-light irradiation.

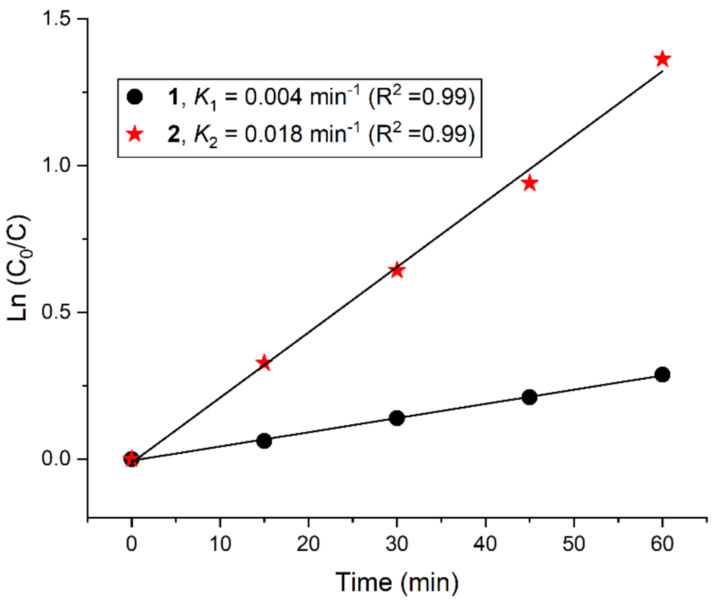

Figure S9. Kinetics of the photocatalytic degradation of TC under visible-light irradiation.

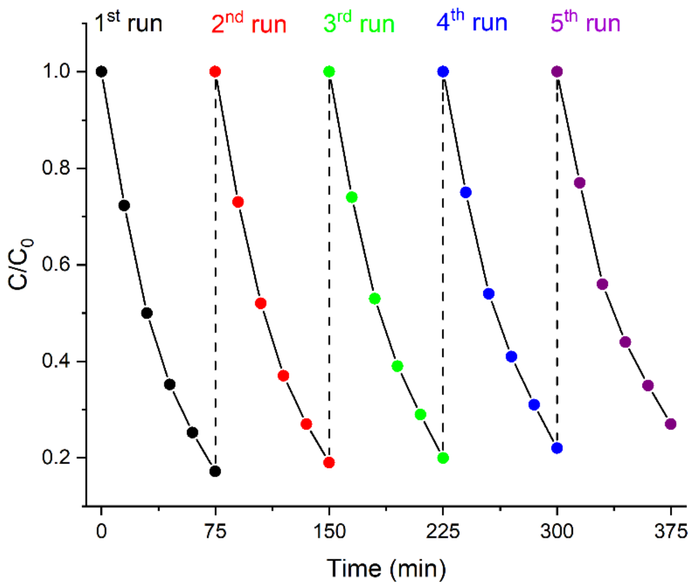

Figure S10. Recyclability of photocatalyst **2** toward the degradation of MO dye.

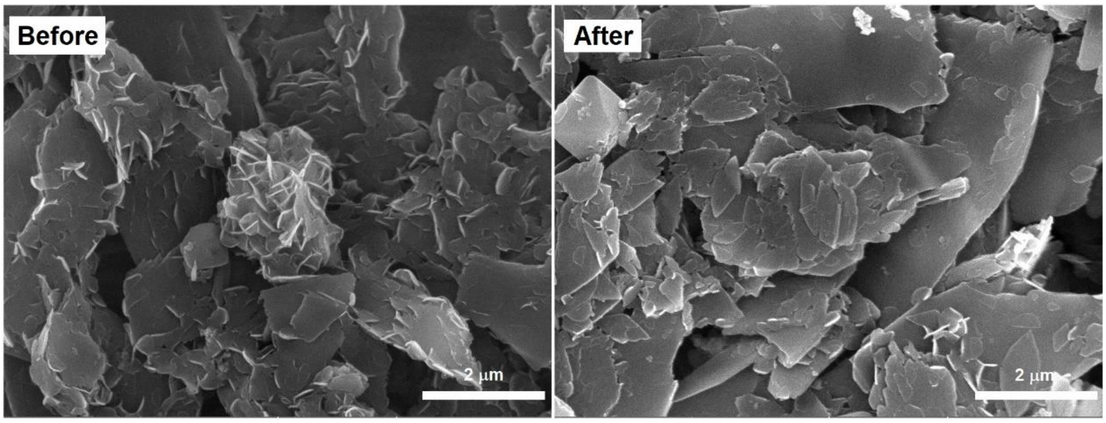

Figure S11. FE-SEM images of **2** (before and after the degradation of MO).

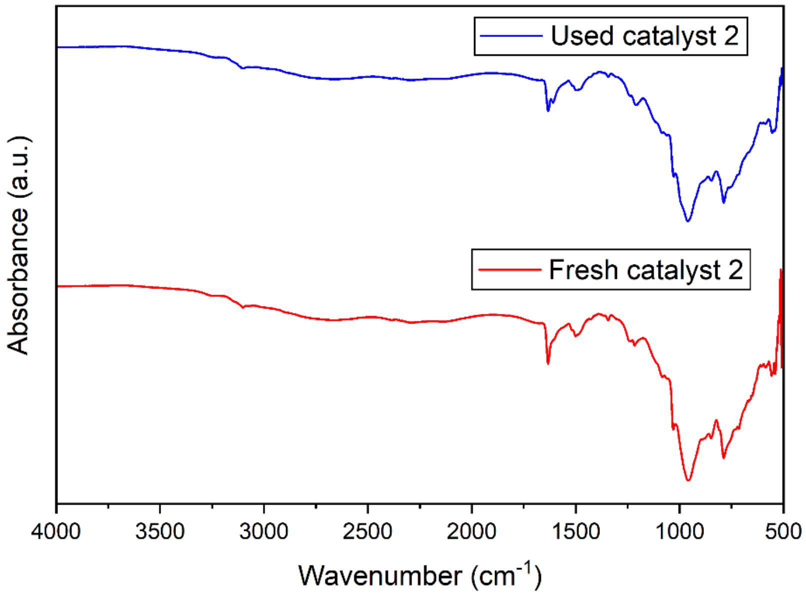

Figure S12. FTIR spectra of **2** (before and after the degradation of MO dye).

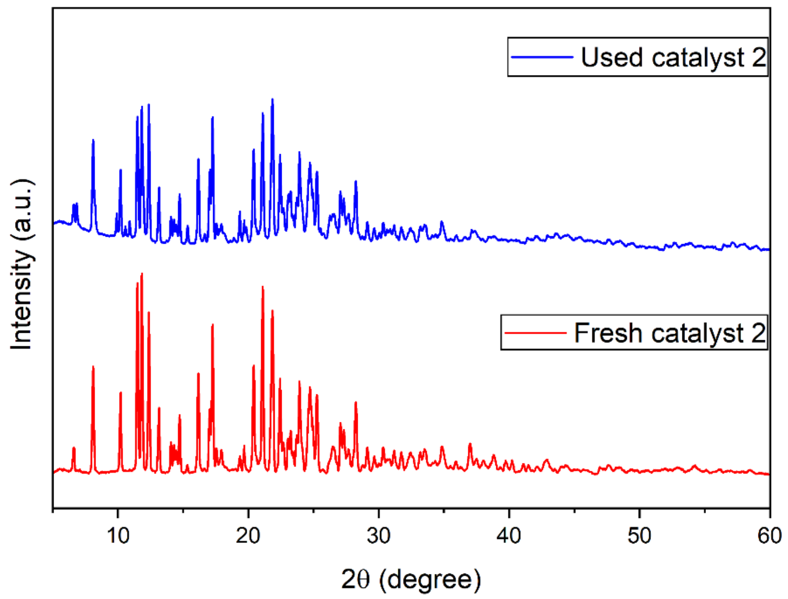

Figure S13. PXRD spectra of **2** (before and after the degradation of MO dye).

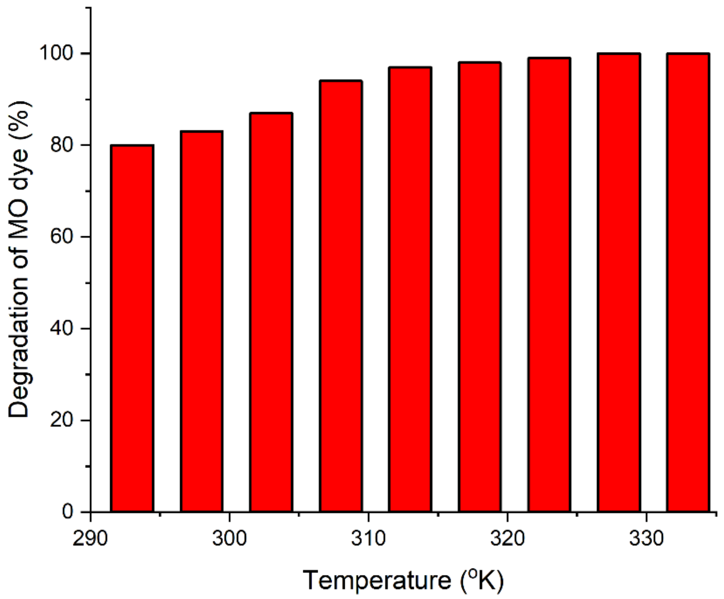

Figure S14. Effect of temperature on the photocatalytic degradation of MO dye in the presence of **2**.

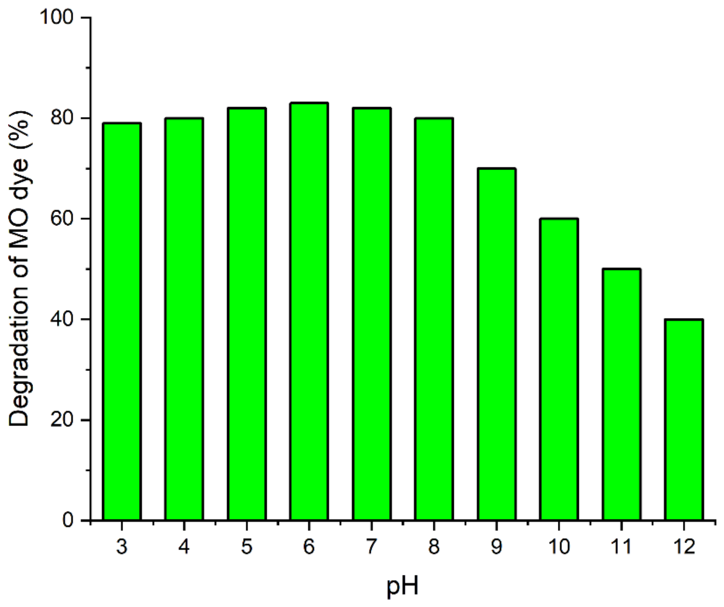

Figure S15. Effect of pH on the degradation of MO dye solution in the presence of **2**.

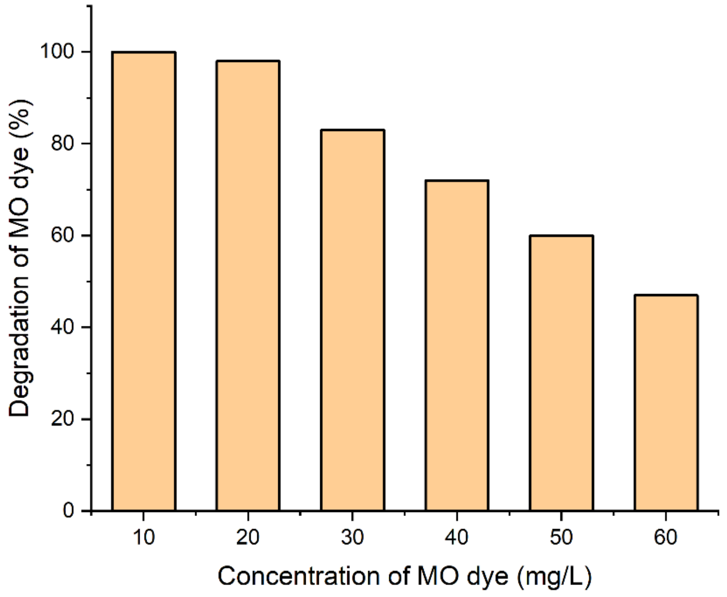

Figure S16. Effect of MO dye concentration on the photocatalytic degradation of MO in the presence of **2**.

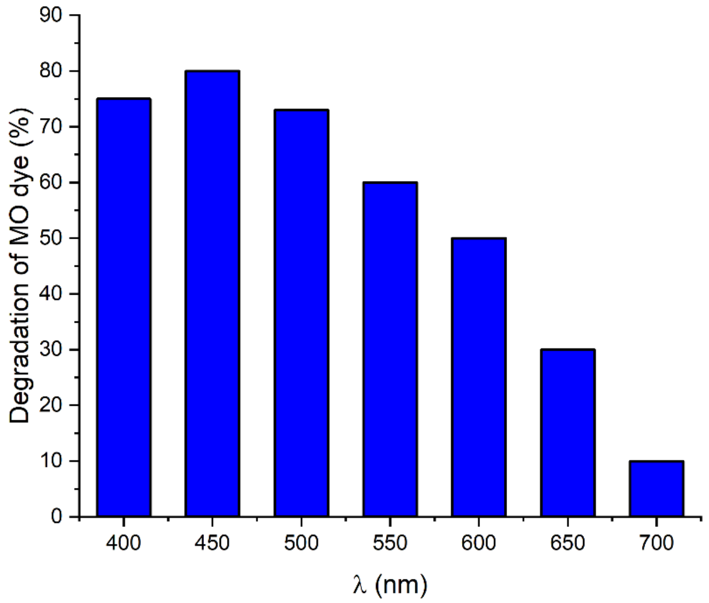

Figure S17. Effect of light intensity on the photocatalytic degradation of MO dye in the presence of **2**.

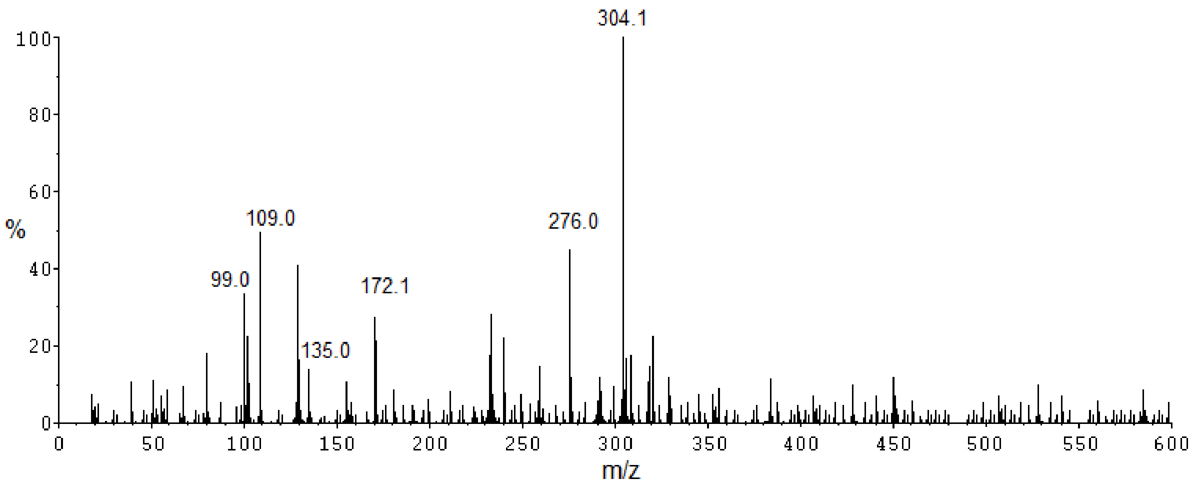

Figure S18. Negative-ion-mode ESI mass spectrum of MO dye degradation catalyzed by **2** after 30 min of visible-light irradiation.

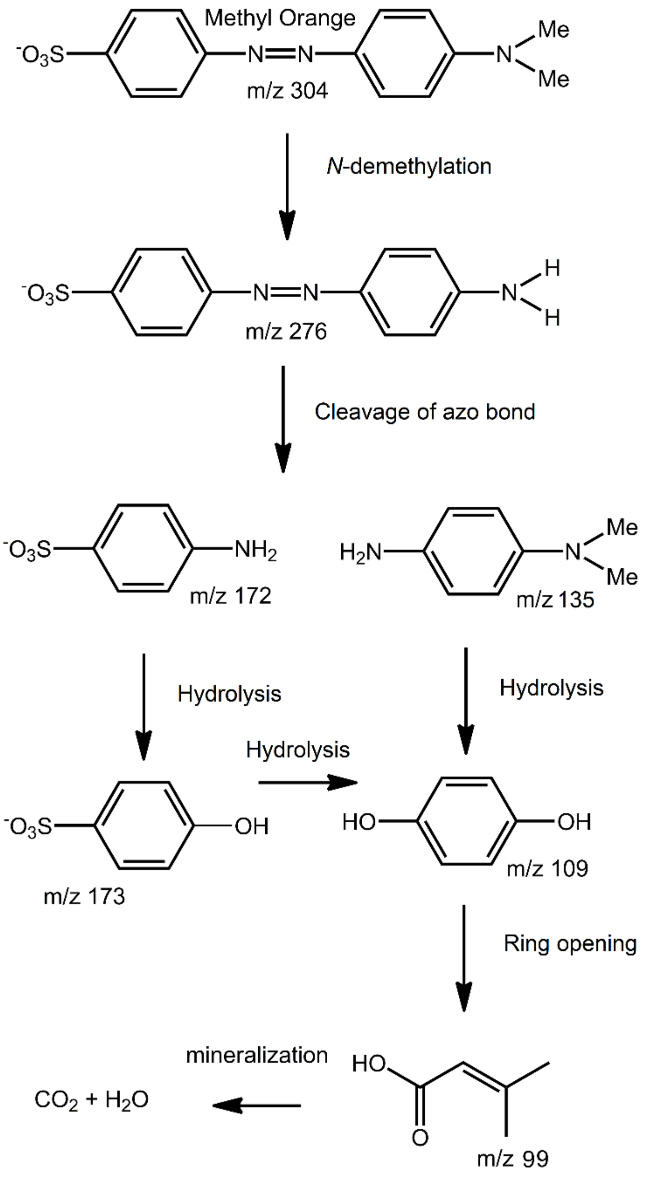

Figure S19. Possible intermediates of the MO dye degradation reaction in the presence of **2** after 30 min of visible-light irradiation.

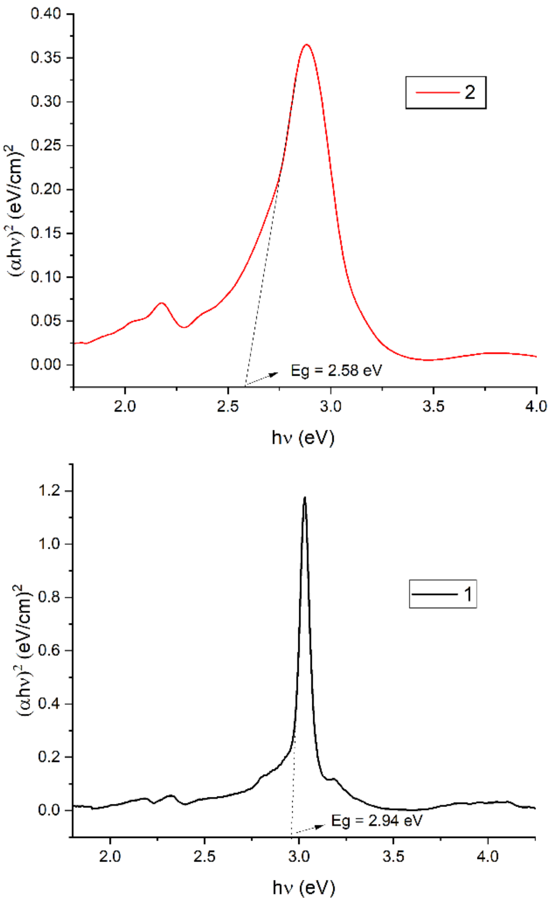

Figure S20. Bandgap energies of **1** and **2** were calculated from the Tauc plots using absorption spectroscopy data.

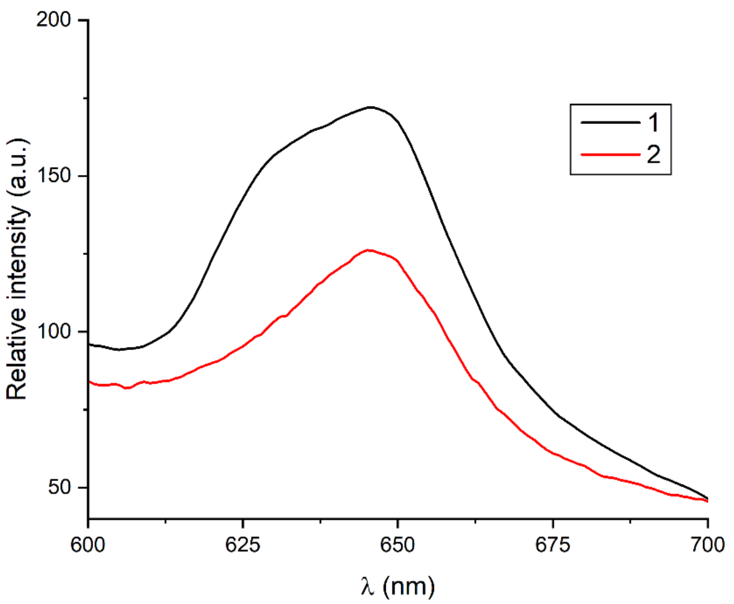

Figure S21. Fluorescence spectra of **1** and **2** in Nujol (C = 10 mg/mL). λ_{ex} = 550 nm.

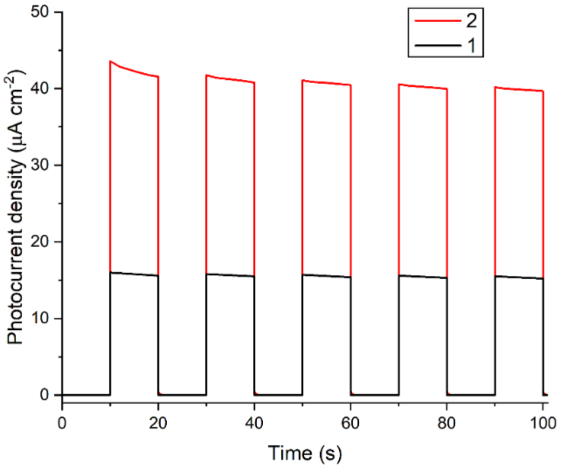

Figure S22. Photocurrent responses for **1** and **2** under visible light.

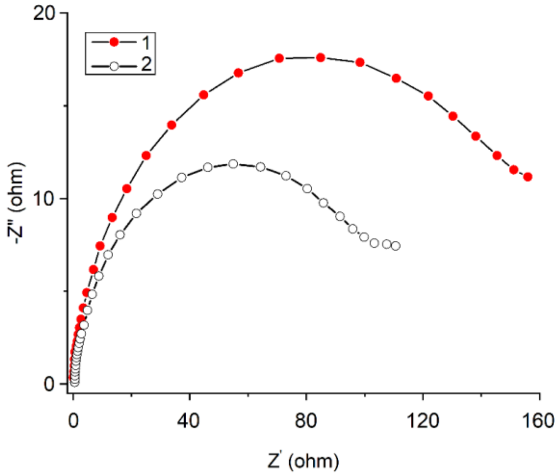

Figure S23. EIS–Nyquist plots of **1** and **2** under visible light.

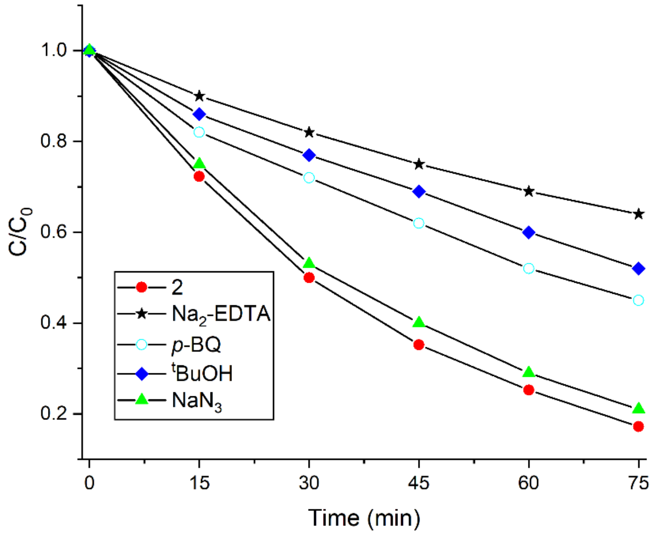

Figure S24. Visible-light MO-dye degradation activities of **2** in the presence of various scavengers.
